# Structural Basis for Inhibitor-Induced Aggregation of HIV Integrase

**DOI:** 10.1371/journal.pbio.1002584

**Published:** 2016-12-09

**Authors:** Kushol Gupta, Vesa Turkki, Scott Sherrill-Mix, Young Hwang, Grant Eilers, Louis Taylor, Charlene McDanal, Ping Wang, David Temelkoff, Robert T. Nolte, Emile Velthuisen, Jerry Jeffrey, Gregory D. Van Duyne, Frederic D. Bushman

**Affiliations:** 1 Department of Biochemistry and Biophysics, Perelman School of Medicine, University of Pennsylvania, Philadelphia, Pennsylvania, United States of America; 2 Department of Microbiology, Perelman School of Medicine, University of Pennsylvania, Philadelphia, Pennsylvania, United States of America; 3 HIV DPU, Infectious Disease Therapy Area Unit GlaxoSmithKline, Research Triangle Park, North Carolina, United States of America; 4 Protein Cellular and Structural Science, GlaxoSmithKline, Collegeville, Pennsylvania, United States of America; Weatherall Institute of Molecular Medicine, UNITED KINGDOM

## Abstract

The allosteric inhibitors of integrase (termed ALLINIs) interfere with HIV replication by binding to the viral-encoded integrase (IN) protein. Surprisingly, ALLINIs interfere not with DNA integration but with viral particle assembly late during HIV replication. To investigate the ALLINI inhibitory mechanism, we crystallized full-length HIV-1 IN bound to the ALLINI GSK1264 and determined the structure of the complex at 4.4 Å resolution. The structure shows GSK1264 buried between the IN C-terminal domain (CTD) and the catalytic core domain. In the crystal lattice, the interacting domains are contributed by two different dimers so that IN forms an open polymer mediated by inhibitor-bridged contacts; the N-terminal domains do not participate and are structurally disordered. Engineered amino acid substitutions at the inhibitor interface blocked ALLINI-induced multimerization. HIV escape mutants with reduced sensitivity to ALLINIs commonly altered amino acids at or near the inhibitor-bound interface, and these substitutions also diminished IN multimerization. We propose that ALLINIs inhibit particle assembly by stimulating inappropriate polymerization of IN via interactions between the catalytic core domain and the CTD and that understanding the interface involved offers new routes to inhibitor optimization.

## Abstract 2

A new crystal structure of the HIV integrase enzyme in complex with the allosteric inhibitor GSK1264 explains how the drug induces aggregation of the viral protein.

## Author Summary

A promising new class of antivirals called “ALLINIs” (allosteric inhibitors of integrase) potently inhibits HIV replication. Like other drugs, ALLINIs seem to target also the HIV-1 integrase (IN), which is crucial for the replication of this virus, but instead of acting at early phases of HIV replication, they interfere with viral particle assembly and maturation that occur at late stages and induce aggregation of IN. Despite these findings, the structural bases for the effects are still unknown. In this study, we crystallized full-length HIV-1 IN in complex with an ALLINI called GSK1264 and determined its structure to 4.4 Å. The structure reveals for the first time the complete ALLINI-binding interface, comprised of both IN C-terminal and catalytic core domains. These domains are contributed from neighboring IN dimers, revealing an open polymeric conformation mediated by inhibitor-bridged contacts. Substitutions at this interface block ALLINI-induced multimerization, and we find that escape mutants against this class of drug lie at or near this interface. We propose that ALLINIs catalyze formation of an open IN polymer, which in turn interferes with viral particle assembly.

## Introduction

Despite the success of antiretroviral therapy for HIV infection, the emergence of drug-resistant viral variants and the recognition of long-term drug toxicities leave development of new drug classes a priority [1]. Integrase strand transfer inhibitors (INSTIs) targeting the active site of the HIV-encoded integrase (IN) protein have proven highly effective [2]. An additional class of IN inhibitors, the allosteric inhibitors of integrase (ALLINIs), act at a second site on HIV IN [3–11]. ALLINIs (also referred to as LEDGINs, noncatalytic site integrase inhibitors [NCINIs], or multimodal inhibitors) are highly active against HIV replication in cell culture but have not yet been fully developed for use in patients, motivating close study to inform ongoing inhibitor development.

**Fig 1:**
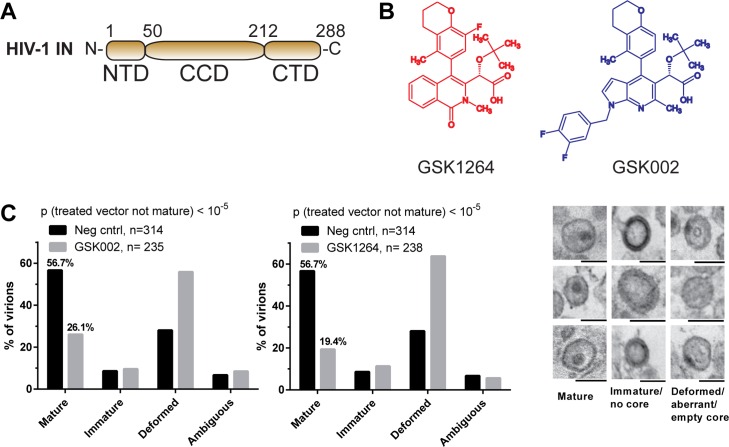
Allosteric inhibitors of HIV IN. (A) Domain organization of IN. (B) Chemical structures of the ALLINIs used in this study. (C) Disruption of assembly by the ALLINIs GSK1264 and GSK002. Viral particles produced in the presence of 1,000 nM GSK1264 or GSK002 were visualized by transmission electron microscopy, and morphology was scored (see S1 Data). The *p*-value is the probability of obtaining the observed (or greater) differences in numbers of nonmature particles (immature, deformed, or ambiguous) between treated and nontreated samples, given the null hypothesis of no inhibitor-induced changes.

During the early steps of HIV infection, IN catalyzes the initial covalent attachment of the viral cDNA to host cell nuclear DNA [12,13]. IN is comprised of three independently folded domains (Fig 1A). The N-terminal domain (NTD; residues 1–50) binds Zn^2+^ via a conserved His-His-Cys-Cys (HHCC) motif. The catalytic core domain (residues 50–212) adopts an RNase H superfamily fold and contains a D,D-35-E motif that binds Mg^2+^ or Mn^2+^ ions, which mediate DNA cleaving and joining. The C-terminal domain (CTD; residues 223–268) features an Src homology domain 3 (SH3)-like fold that contributes to DNA binding and is connected to the catalytic core domain by a α-helical linker (residues 213–222).

IN function is assisted by the cellular cofactor lens epithelium-derived growth factor (LEDGF/p75), which binds tightly to a site at the catalytic core domain dimer interface [12,13]. LEDGF/p75 binding targets HIV integration to active transcription units [14–16] and promotes the integration reaction [12,13,17]. These findings and the observation that small molecules can bind this site [4,10] motivated screens for small molecules that block LEDGF/p75 binding to the catalytic core domain. Multiple compounds were identified and shown to bind the expected site on the catalytic core domain by X-ray crystallography, and many showed potent antiviral activity in cell culture [3,4,7–9,11,18,19]. However, mapping the target of ALLINI inhibition within the HIV replication cycle revealed a surprise: ALLINIs interfered only modestly with early steps of HIV replication but potently disrupted late steps, including particle assembly and maturation [6–9,18]. As a result, defective particles were produced with abnormal organization of the viral RNA and nucleocapsid (NC) proteins and greatly reduced infectivity [6,8]. It has been unclear how binding of ALLINIs to the IN catalytic core domain could interfere with particle maturation.

Here we report the crystal structure of HIV IN bound with the ALLINI GSK1264 at 4.4 Å. Prior to this study, atomic insight into the interaction of GSK1264 with IN was limited to the catalytic core domain only [7], a result which did not completely explain the mode of action of this class of compound. Here we report that the complete GSK1264 binding interface is comprised of both the CTD and the catalytic core domain, which together almost entirely bury the compound within protein. The interface is rich in residues implicated in IN oligomerization and ALLINI sensitivity, indicating likely functional significance. The dimer–dimer interaction mediated by GSK1264 leads to formation of an open polymer in the crystal, a polymerization event that is readily reproduced in solution with purified components and readily attenuated by mutagenesis. To probe ALLINI function more broadly, we compared the properties of ALLINIs in biochemical, virological, and electron microscopic assays. Several IN variants, including escape mutations elicited by growth of HIV in the presence of ALLINIs, encode IN substitutions at or near the inhibitor binding site, and these substitutions also resulted in decreased IN oligomerization in vitro. The results support a mechanism in which ALLINIs disrupt viral particle maturation by promoting formation of IN polymers, as in the IN-GSK1264 crystal structure. Identification of the molecular interface responsible for polymer formation establishes a structural basis for improving the ALLINI class of HIV inhibitors.

## Results

### GSK1264 and GSK002 Disrupt Formation of Mature HIV Particles

To investigate the structural basis for ALLINI function, we studied GSK1264 (described in [7]) and GSK002, which is newly reported here (Fig 1B). We first examined whether these compounds disrupt HIV particle organization, as has been documented for other ALLINIs [6,8,18], by comparing the viral particles produced from infected cells using transmission electron microscopy (Fig 1C). Inhibitor exposure significantly reduced the percentage of mature virions with proper morphology compared to untreated control cultures (100 nM and 1,000 nM doses, *p*-values < 10^−5^). This result is consistent with the potent effect of GSK1264 late in the viral replication cycle [7] and parallels results for other ALLINIs [6–9,18].

### Structure of an HIV IN•GSK1264 Complex

To crystallize HIV IN bound to an ALLINI, we sought substitutions that improved IN solubility but preserved some degree of ALLINI-dependent IN aggregation, allowing control of IN self-association. We used size-exclusion chromatography in line with multiangle light scattering (SEC-MALS) and analytical ultracentrifugation (AUC) to establish the solution properties of several IN variants. Our best candidate contained two amino acid substitutions: Y15A and F185H. IN^Y15A,F185H^ formed discrete dimers in solution, retained the ability to bind to the LEDGF integrase binding domain (IBD), and had a reduced level of aggregation in the presence of GSK1264 and GSK002 compared to wild-type IN (S1 Fig and S1 Table). Substitutions of F185 are known to improve solubility [20], and viruses containing the F185H substitution are replication competent [21]. The Y15A substitution selects for a single conformation of the isolated NTD [22] that is correlated with diminished oligomerization of full-length IN [23]. We favored NTD substitutions for this study because IN constructs containing only the catalytic core domain and the CTD were previously shown to be sufficient for ALLINI-induced aggregation [7,24], suggesting that NTD substitutions would not interfere with understanding ALLINI action. HIV-1 containing IN^Y15A^ is replication defective, and the purified protein is not active for integration.

**Fig 2:**
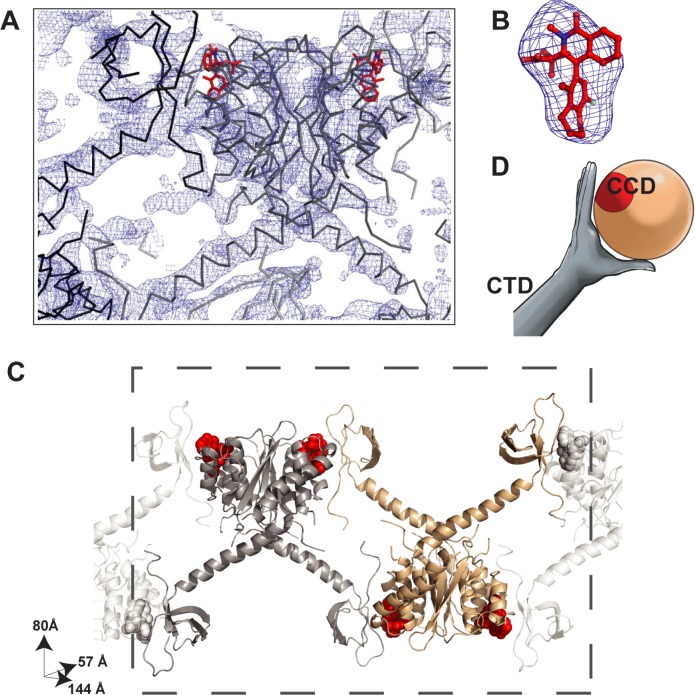
Crystal structure of HIV-1 IN^Y15A,F185H^•GSK1264 complex. (A) Shown is a simulated annealing composite-omit 2F_o_-F_c_ electron density map contoured to 1.5 σ (shown in blue). Contiguous electron density is observed for the catalytic core domains, the CTDs, and GSK1264 (red), but the NTDs are disordered. (B) Simulated annealing F_o_-F_c_ map density for GSK1264, contoured to 3σ (shown in blue). (C) The ALLINI-bound HIV-1 IN polymer observed in crystals. One dimer of HIV-1 IN comprises the asymmetric unit. Bound GSK1264 is shown by the red spheres, two interacting dimers are highlighted in the dotted box, and additional subunits of the open polymer are shown in light grey. (D) Cartoon of the CTD–catalytic core domain interaction, where the hand represents the CTD, the ball represents the catalytic core domain dimer, and GSK1264 is shown in red. See also S2 Fig and S2 Table.

Crystallization trials of IN^Y15A,F185H^ with GSK1264 led to a diffracting crystal form containing one IN dimer in the asymmetric unit; trials with GSK002 did not yield crystals. We determined the structure by molecular replacement using the catalytic core domain dimer structure (without ligand) from the GSK1264-bound structure previously determined (PDB 4OJR, [7]) together with the previously reported IN CTD structure [7,25]. The structure was refined using deformable elastic network (DEN) restraints [26–28]. The catalytic core domains, CTDs, and connecting linkers displayed strong electron density in simulated annealing 2F_o_-F_c_ composite omit maps, and thus, their positions are well defined in the structure (Fig 2A). The IN NTDs could not be positioned by molecular replacement and did not display interpretable electron density. However, IN recovered from washed crystals was full length by SDS-PAGE (S1 Fig). Hence, the NTDs are presumed to be spatially disordered. Strong difference density in simulated annealing omit maps was observed at both previously observed GSK1264 binding sites on the catalytic core domain dimer [7], allowing for placement of the ligand in the latest stages of refinement (Fig 2B). At the modest resolution available for this crystal form, we were able to reliably place domains, secondary structure elements, and the bound inhibitor, but we could not visualize detailed side chain interactions. We inferred the approximate positions of side chains from the electron density and their known locations in the high-resolution crystal structures of smaller IN fragments [7,29]. Diffraction data and refinement results are provided in S2 Table.

The GSK1264-bound HIV IN structure is shown in Fig 2C. The IN CTDs bind to the catalytic core domains and bound inhibitors in adjacent IN dimers, resulting in the formation of an open polymer of IN dimers. The interaction site resembles a hand (the CTD) grasping a ball (the catalytic core domain), with the ALLINI site cupped by the fingers (Fig 2D).

### The GSK1264–IN Binding Interface

**Fig 3:**
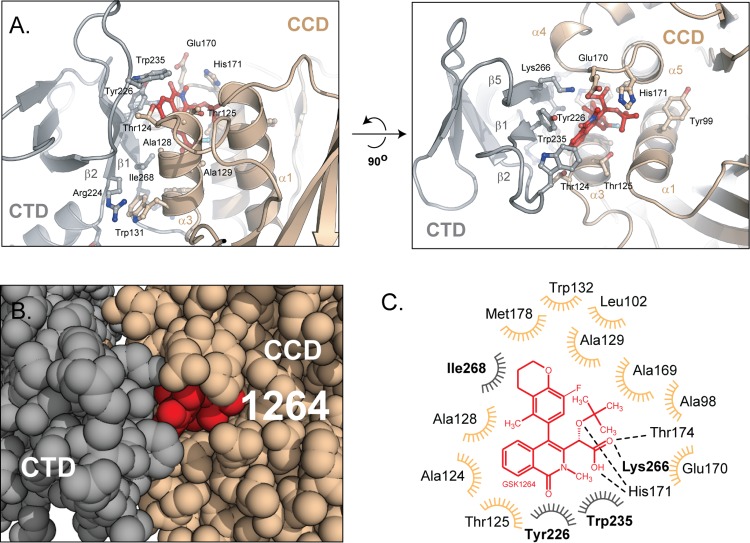
The GSK1264 binding interface. (A) Orthogonal views of the GSK1264-induced polymer interface. GSK1264 is shown in red, packed between the CTD (grey) and the catalytic core domain dimer (tan). The positions of the side chains rendered are inferred from the high-resolution crystal structures used in the DEN refinement procedure. (B) Sphere rendering of the protein–ALLINI-protein interface. Colored as in A. (C) Schematic of IN-GSK1264 contacts. GSK1264 (red) is predominantly buried via van der Waals contacts with 13 residues from the CTD (grey) and catalytic core domain (tan). Thr^174^, Lys^266^, and His^171^ are predicted to hydrogen bond with the *tert*-butoxy and carboxylic acid moieties. This panel was generated using LIGPLOT [30]. See also S2 Fig, S3 Fig and S1 Table.

In the IN•GSK1264 complex, GSK1264 is almost entirely protected from solvent, with 500 out of 580 Å^2^ of its accessible surface buried (Fig 3A–3C). Excluding bound inhibitor, over 1,100 Å^2^ of solvent-accessible surface is buried at each CTD–catalytic core domain interface. Secondary structure elements mediating GSK1264 binding include one face of the CTD, defined by strands β1, β2, and β5, juxtaposed against the catalytic core domain dimer near helix α3 of one subunit and α4 of the other. The CTD–catalytic core domain interface has a strong electrostatic component, with the relatively acidic α4 helix of the catalytic core domain packing against the basic β5 strand of the CTD (S2 Fig). Extensive hydrophobic interactions are also formed between α3 of the catalytic core domain and the β1 and β2 strands of the CTD.

The modest resolution of our structural model precludes direct visualization of interactions but does allow us to model the approximate positions of functional groups and potential contacts mediating GSK1264 binding (Fig 3A and 3C). The Tyr^226^ and Trp^235^ side chains in the CTD pack against the core isoquinoline moiety, where π–π interactions between inhibitor and aromatic side chains may contribute to binding stabilization. The *tert*-butoxy moiety is buried in a hydrophobic pocket formed by the catalytic core domain dimer, without contributions from the CTD. The benzopyran group extending from the isoquinoline core is surrounded by a cradle of hydrophobics at the catalytic core domain dimer interface and, additionally, Ile^268^ from the CTD. A modeled hydrogen-bonding network involving the carboxylate and *tert*-butoxy oxygen completes the IN-inhibitor interface, where Lys^266^ from the CTD is positioned to coordinate the carboxylate moiety. S2C Fig shows a surface model of interactions between each domain and GSK1264.

### Functional Analysis of the ALLINI-Binding Interface

**Fig 4:**
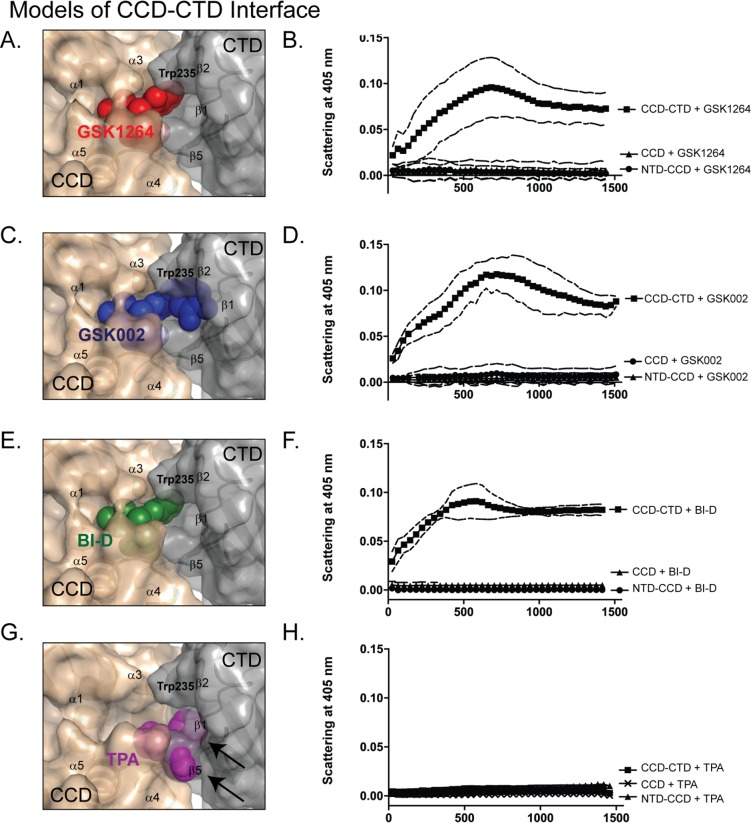
Experimental probing of the GSK1264 interface. Models of the catalytic domain–CTD interface with bound small molecules (A, C, E, and G) are shown beside aggregation time course assays for the same compounds (B, D, F, and H). Aggregation assays contained one and two-domain fragments of IN^F185H^ as indicated (“CCD” denotes catalytic core domain). Compounds studied are GSK1264 (A, B; red, PDB 4OJR [7]), GSK002 (C,D; blue, PDB 5HRN [this work]), BI-D (E, F; green, PDB 4ID1 [8]), and tetraphenylarsonium (TPA; G, H; magenta, PDB 1HYV [10]). Models were generated by docking the indicated CCD/compound structures into the GSK1264/ IN^F185H^ structure studied here. Aggregation assays were carried out using light scattering at 405 nm at 25°C with 10 μM IN (see S1 Data). In panel G, arrows indicate steric clashes predicted by modeling of the CTD–catalytic core domain interface with TPA, based on the TPA binding mode in PDB 1HYV [10].

To probe the importance of the GSK1264 interface observed in this crystal form, we compared the IN aggregation properties and structural features of several compounds bound at ALLINI sites (Fig 4). Previously, we and others have reported [7,24] that truncated IN derivatives containing only the catalytic core domain and the CTD were sufficient to support aggregation by ALLINIs in vitro. In addition, crystal structures are available for the IN catalytic core domain bound to several ALLINIs and other small molecules (Fig 4A–4D). Structures of the GSK1264 [7], BI-D [8], and tetraphenylarsonium (TPA) [10] complexes were reported previously; the structure of GSK002 bound to the catalytic core domain at 1.75 Å is newly reported here (Fig 4C, S2 Table, and S4 Fig). We found that GSK1264, GSK002, and BI-D all directed potent aggregation of IN containing the catalytic core domain and the CTD, but not IN derivatives containing the NTD and the catalytic core domain or only the catalytic core domain (Fig 4). TPA, which is not an ALLINI, did not promote aggregation of any derivative even at millimolar concentrations (Fig 4H).

Analysis of the TPA-bound catalytic core domain structure superposed onto the GSK1264-bound full-length IN structure indicates that TPA would clash with the CTD and therefore block the CTD–catalytic core domain interaction seen for GSK1264 (arrows in Fig 4G). A small steric clash is also predicted for GSK002, in which the difluorobenzyl group collides with Trp^235^. However, rotation of the Trp^235^ side chain could create enough space for the inhibitor, so we suggest that GSK002 could be accommodated in a nearly identical interface. Thus, this analysis supports the concept that CTD–ALLINI–catalytic core domain interactions are relevant to aggregation in vitro and that the shape and positioning of the ALLINI molecule is critical for the aggregation reaction.

### CTD Substitutions That Block ALLINI-Induced Aggregation

**Fig 5:**
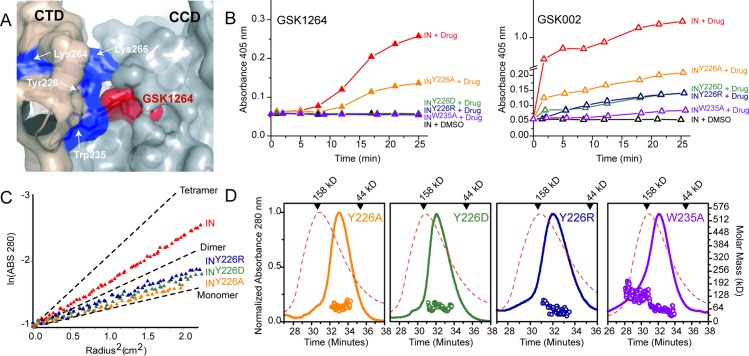
CTD substitutions affect IN oligomerization and ALLINI-induced aggregation. (A) CTD residues (shown in blue) at the IN^F185H^–ALLINI interface mapped onto the IN^F185H^–GSK1264 structure. GSK1264 is shown in red. Mutations at K264 and K266 that affect ALLINI function were previously reported [24]; the effects of mutations at Y226 and W235 are reported here. (B) Aggregation assays. The time-dependent aggregation of IN by GSK1264 and GSK002 was monitored using absorbance optics. To initiate the reaction, either DMSO or drug was added to recombinant IN^F185H^ for final concentrations of ~40–61 μM IN^F185H^ monomer and 44 μM drug at room temperature. For each panel in this figure, “IN” denotes “IN^F185H^”. (C) Analytical ultracentrifugation sedimentation equilibrium analysis. Data were recorded at 12,000 RPM, at a concentration of ~10 μM IN^F185H^, at 4°C. Linearized radial distributions are shown. The slopes are proportional to M_w_ at a given value of r^2^. Single-species plots with calculated slopes for idealized IN^F185H^ monomer, dimer, and tetramer are shown for the same rotor speed and temperature as black lines. (D) SEC-MALS analysis. The IN^F185H^ SEC trace is superimposed on each panel in the dotted red line. Retention times for molecular weight markers are shown at the top of each panel, and retention times for globular molecular weight standards are shown as open circles. Elution concentrations by refractive index approached ~0.1 mg/mL. Experiments were performed at room temperature using a Superdex 200 10/300 column. Data shown in panels B–D are provided in S1 Data.

IN substitutions that diminish ALLINI function have been studied on the catalytic domain side of the interface, but interactions on the CTD side are less well characterized. Biochemical studies have implicated the CTD in IN oligomerization [7,20,31,32], and CTD residues K264 and K266, when both mutated to alanine, diminished ALLINI-induced aggregation and blocked integration activity [24]. In the structure presented here, these residues contribute to the GSK1264 interface (Fig 5A), supporting the biological relevance of the observed contacts. We further probed the importance of this interface by studying substitutions at CTD residues Y226 and W235 (S1 Table, Fig 5 and S5 Fig), which are large hydrophobics comprising a substantial portion of the surface buried. Substitutions at Y226 or W235 inhibited precipitation by GSK1264 and GSK002 (Fig 5B) and abrogated IN activity (S5 Fig). Three substitutions at Y226 each resulted in IN derivatives that showed diminished oligomerization in vitro by both AUC (Fig 5C) and SEC-MALS (Fig 5D). Purified IN W235A also showed diminished oligomerization by SEC-MALS (Fig 5D) but was too unstable for sedimentation equilibrium analysis. In summary, four CTD residues in the ALLINI interface are required for ALLINI-induced aggregation of IN, supporting the biological importance of the interface shown in Fig 2, Fig 3 and Fig 5A.

### Mechanism of GSK1264 and GSK002 Resistance Mutations

To identify mutations that confer resistance to GSK1264 and GSK002, we passaged HIV-1 in the presence of increasing concentrations of each inhibitor and isolated resistant viral strains. Three different HIV-1 strains were compared for each inhibitor: the lab-adapted HIV strain NL4-3 and Raltegravir-resistant strains 4376 and 8070. Strains were passaged 38 times in the presence of compound, resulting in an increase in the inhibitory IC_50_ of >100-fold. IN coding regions were sequenced, and departures from wild-type sequence tabulated (S3 Table).

**Fig 6:**
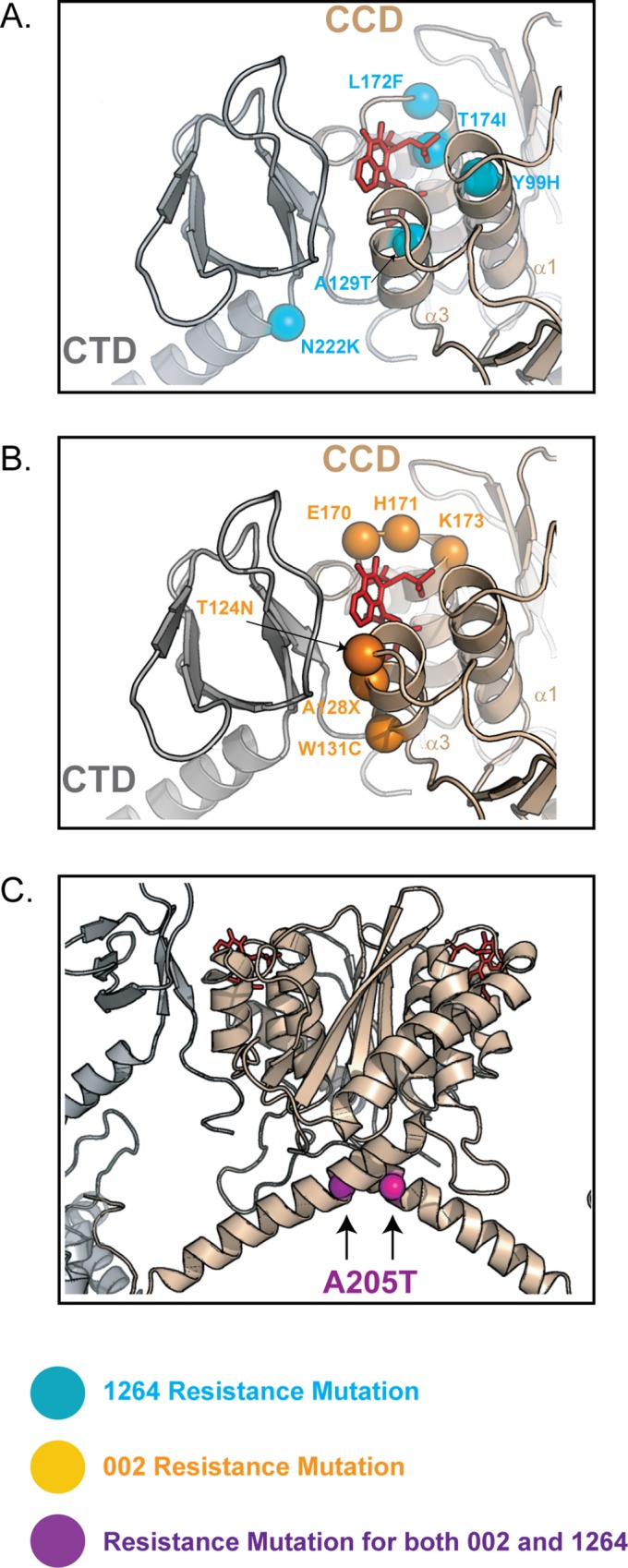
HIV IN mutations conferring reduced sensitivity to GSK1264 and GSK002 after serial passage. (A) Amino acid substitutions resulting from resistance mutations identified by serial passage in the presence of GSK1264 (blue spheres). (B) Substitutions identified in the presence of GSK 002 (orange spheres). Bound GSK1264 is shown in red. (C) The A205T resistance substitutions, which arose in both experiments. S4 Fig provide structural insights into the mechanism of resistance conferred by polymorphisms at residues 124 and 125.

Each of the three viral strains studied showed mostly distinct escape mutations that were unique to each inhibitor (Fig 6). Multiple independent viral cultures were not compared for each ALLINI, so it is unknown whether the screens were saturated. Some of the substitutions match previously published ALLINI resistance mutations [3–5,9,11,18,19,33–35]. The IN•GSK1264 structure allows us to consider the mechanism by which these escape mutations confer resistance.

In studies with GSK1264, six different substitutions were found (Fig 6A and S3 Table). Ala^129^ and Thr^174^ are positioned to contact GSK1264 directly, so the larger side chains seen in escape mutants (A129T and T174I) may disrupt inhibitor binding via steric clashes. Y99H and L172F do not contact bound GSK1264 but may disrupt inhibitor binding via changes in occupied volumes within the catalytic core domain, resulting in displacement of α1 (Y99H) or α5 (L172F). Ala^205^ and Asn^222^ lie at the beginning and the end of the α-helical linker that bridges the catalytic core domain and the CTD of IN, so substitutions may alter the CTD position relative to the catalytic domain, thereby potentially inhibiting formation of the ALLINI complex (Fig 6A and 6C).

Eight mutations arose after passage in the presence of GSK002, only one of which was in common with the GSK1264 mutations (Fig 6 and S3 Table). Using the GSK1264 structure as a model, we can propose mechanisms of GSK002 resistance for several of these. IN residues 125, 128, 170, 171, and 173 are poised to contact the bound inhibitor and/or the CTD, so substitutions at these positions likely disrupt the inhibitor-mediated interface directly. A205T was seen for both GSK002 and GSK1264 resistance substitutions (described above). T124N was the only substitution to arise independently in two experiments (S3 Table), and substitutions at this position and at residue 128 have been reported as ALLINI resistance mutations previously [3–5,11,19,33–36].

Ala^124^ and Trp^131^ are the first and last residues of the α3 helix; they contribute to the oligomerization interface between the catalytic core domain and the CTD, so substitution of these residues is likely to interfere with inhibitor-mediated oligomerization. The resistance mutation encoding W131C was identified in the earliest passages and is unique to this study (Fig 6B and S3 Table). W131D-substituted IN has been reported previously to increase solubility of purified IN [29,37,38]. Trp^131^ is largely buried in the CTD interface in the IN•GSK1264 structure reported here (S3 Fig). Trp^131^ does not contribute to the ALLINI binding interface directly, but the IN^W131D^ substitution shows reduced oligomerization and reduced sensitivity to ALLINI-induced aggregation (S3 Fig and S1 Table), supporting the idea that the Trp^131^–CTD interaction is important for the inhibitor response.

To assess cross-resistance between GSK1264 and GSK002, we examined the sensitivities of two long-term passaged variants. Thirty-eight passages in increasing concentrations of GSK002 yielded a variant with T124N, W131C, and A205T; 38 passages in GSK1264 yielded a variant with Y99H, L172F, and N222K. In both cases, these multiply mutant variants were highly resistant to both drugs (>550-fold increase in IC_50_).

To investigate the mechanism of ALLINI resistance in vitro, we purified six IN variants with escape mutant substitutions and tested their oligomeric properties using sedimentation equilibrium. Each of the escape variants showed reduced oligomerization compared to IN^F185H^, suggesting that diminished multimerization is a common characteristic of ALLINI escape substitutions (S1 Table).

We also purified IN variants containing resistance substitutions along the α3 helix and tested their ability to be aggregated by GSK1264 or GSK002 (S1 Table). For IN^F185H^, 0.8 μM of GSK002 or 8 μM of GSK1264 was sufficient to promote aggregation. The T124N, A128T, and W131C substitutions each protected IN from aggregation by either compound at 67 μM. The T124A substitution protected IN from polymerization at 67 μM GSK1264, but not GSK002. These findings strengthen the link between HIV escape from ALLINI pressure in cell culture and diminished inhibitor-induced polymerization of IN in vitro.

### Sequence Polymorphisms Affecting Sensitivity to ALLINIs

**Fig 7:**
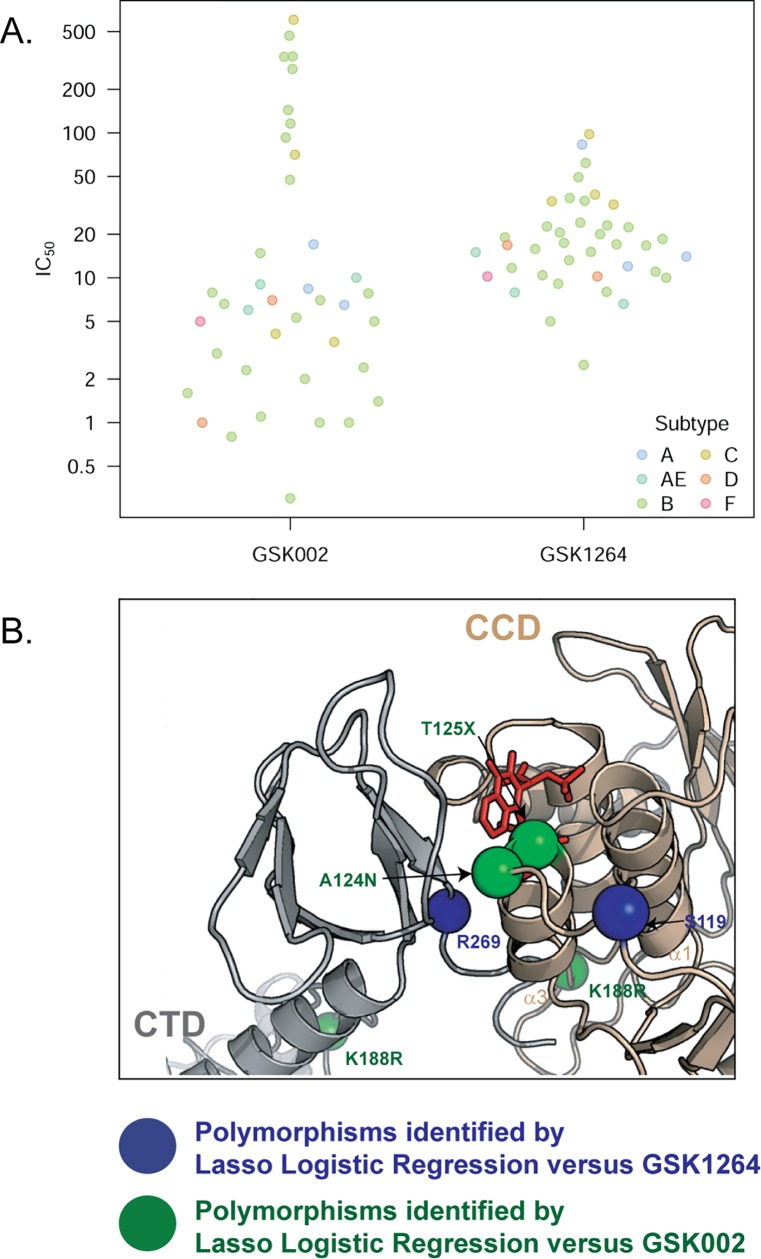
Sensitivity of HIV strains to GSK1264 and GSK002. (A) Distributions of IC_50_ values for GSK1264 and GSK002 in infections of multiple subtypes of HIV, colored by subtype and sorted by small molecule. The *y*-axis shows IC_50_ values. Data are provided in tabular form in S1 Data. (B) Polymorphisms identified by lasso logistic regression linked to resistance to GSK1264 (blue spheres) or GSK002 (green spheres) mapped onto the IN-GSK1264 structure. GSK1264 is shown in red. See S3 Table.

To characterize naturally occuring IN variants that affect ALLINI activity, we tested a panel of 38 HIV isolates for sensitivity to GSK1264 and GSK002. Viruses of subtypes A, B, C, D, F, and A/E were compared, thereby interrogating a wide range of primary IN amino acid sequences. For GSK1264, the IC_50_ values ranged from 2–100 μM (Fig 7A). In contrast, IC_50_ values determined for GSK002 against the same panel ranged from less than 0.5 μM to greater than 500 μM (Fig 7A). There was little correlation among IC_50_ values for the two inhibitors (R = 0.0492, R^2^ = 0.0024, *p* = 0.811), emphasizing the differential effects IN sequence variation can have on the potency of ALLINIs.

The amino-acid polymorphisms best able to predict IC_50_ were extracted from the data for each inhibitor using lasso logistic regression [39]. A model predicting GSK002 activity using the top four most influential amino acid substitutions (K20R, A124N, T125X, where X ≠ T, and K188R) performed well under cross validation, where the cross-validated mean-squared error decreased from 8.49 to 2.26. The model for GSK1264 showed weak predictive power, with no mean-squared error greater than one standard error away from random. Substitutions at Lys^20^, Ala^23^, Ser^119^, and Arg^269^, selected earliest by lasso logistic regression, may have the potential to influence IC_50_ for GSK1264, though less robustly than for residues affecting GSK002.

Positions of affected residues near the inhibitor interface are shown on the IN•GSK1264 structure in Fig 7B. Substitutions at residue 124 could affect ALLINI binding or the catalytic core domain–CTD interaction. Substitutions at 125 may also affect ALLINI binding. Arg^269^ lies on the CTD at the interface with the IN catalytic core domain adjacent to the ALLINI binding site, where substitutions could disrupt this interaction. We consider the possible roles of additional substitutions, including those in the NTD (K20R, A23X), in the Discussion.

### Probing the Mechanism of Resistance to GSK002

To investigate the mechanism of GSK002 resistance further, we determined three high-resolution crystal structures, one of GSK002 bound to catalytic core domain-only, one of GSK002 bound to the catalytic core domain containing the Ala^124^ substitution, and one of GSK002 bound to the catalytic core domain with Thr^125^. These two substitutions were chosen for study because they were the most influential in reducing GSK002 IC_50_ in the study of diverse HIV isolates (Fig 7, S3 Table and S4 Fig). The protein structures were quite similar, with RMSDs ranging from 0.09 to 0.13 Å in pairwise comparisons (S4 Fig). However, the azaindole core of the bound GSK002 ligand is seen to shift in position by ~0.5 Å, depending on the identity of the substitutions at residue 125, along with movement of the difluorobenzyl group by almost 1 Å (S4A–S4C Fig). Overlay of the catalytic core domain•GSK002 structures on the IN•GSK1264 structure suggests a further mechanism of resistance. Larger substitutions at residue 124 and 125 would create a steric clash with the polypeptide backbone of the IN CTD, disrupting the inhibitor-mediated interaction (S4D Fig). Thus, the larger side chains in the resistance substitutions appear to either clash directly with the IN CTD or displace the bound inhibitor sufficiently to result in an inhibitor clash with the CTD.

## Discussion

Here we have identified the determinants of IN aggregation mediated by ALLINIs and obtained a structural model of the specific interfaces involved. In our model, IN polymers are formed by catalytic core domain–CTD interactions that are facilitated and strengthened by the bound ALLINI. We propose that ALLINIs act by causing IN to form extended polymers mediated by this interaction, which in turn leads to aggregation and inactivation. This mode of inhibition is reminiscent of microtubule-stabilizing agents such as Taxol that stimulate lateral interactions between oligomers that modulate assembly [40,41]. Our model is supported by the properties of IN mutants that are resistant to ALLINIs, including mutants elicited during HIV passage in the presence of ALLINIs, engineered INs, and naturally occurring IN polymorphisms in circulating HIV strains. Our model is also supported by a strong correlation between in vivo inhibition and susceptibility to polymerization of purified IN proteins in vitro.

Why was the catalytic core domain–CTD interaction seen here not observed in previous experimental structures of HIV IN? We suggest that the answer lies with the use of (3-((3-cholamidopropyl) dimethylammonio)-1-propanesulfonate) (CHAPS), the zwitterionic detergent used for decades to solubilize IN for in vitro experiments [29,42]. CHAPS binds to the same CTD surface used to form ALLINI-mediated polymers, providing an explanation for how the detergent prevents aggregation and for why previous structural studies would not have revealed the intermolecular interfaces reported here (S2 Fig).

How does aggregation of IN disrupt viral assembly? ALLINIs induce aggregation of IN in vitro, and escape mutations reverse this, implicating aggregation in the mechanism of inhibition. During assembly, IN is first synthesized as part of the Gag-Pol polyprotein precursor. After encapsidation, Gag-Pol is cleaved into component proteins, including IN [12,13]. Immature virions containing Gag-Pol formed in the presence of ALLINIs appear normal in electron microscopy (EM) images, but mature particles are abnormal, suggesting that IN, and not Gag-Pol, may be the ALLINI target. This model is consistent with studies in which ALLINIs were effective when IN was delivered to viral particles independently of Gag-Pol [6,8]. HIV particles can bud properly when Gag only is present to yield immature particles, but Gag-PolΔIN forms defective particles during maturation [6]. The defective particles have electron-dense material outside the normal core, as is seen after ALLINI treatment, reportedly containing NC and RNA [6]. Thus, the IN polymer seen here could act by either (1) sequestering IN and preventing a normal function important for localizing NC and the RNA during maturation or (2) creating a novel aggregate that is itself disruptive.

IN substitutions that confer resistance to ALLINIs in virus fall into three categories, the first of which likely interfere directly with inhibitor binding (e.g., A128T, E170K, and H171T). The second group perturb the catalytic core domain–CTD interaction that mediates polymer formation (e.g., A124N). These substitutions can lie at the interface between domains (e.g., K266A and W131C/D), in a “second shell” that flanks the binding site (e.g., Y99H and L192F), or in the helical connector between domains that is required for positioning the CTD (e.g., A205T and N222K). While mutations at 226, 235, 264, and 266 all confer resistance to ALLINI-induced aggregation in vitro, no strong escape mutants at the CTD side of this interface occur naturally. However, most of the residues on the CTD that are in contact with GSK1264 are absolutely conserved among HIV variants, suggesting that substitutions that block ALLINI binding are likely to be replication defective. The third group of ALLINI-resistance substitutions resides within the NTD (e.g., D6R, E11K, and Y15A). These substitutions are located on a face of the NTD that has been observed bound to the catalytic core domain in several reported crystal structures [23,38,43,44]. At present, we favor a model in which binding of the NTD to the catalytic core domain competes with binding of the CTD. Enhanced binding of the NTD as a result of amino acid substitutions could inhibit ALLINI-promoted engagement of the CTD at the catalytic core domain. Further work will be required to understand how these NTD substitutions modulate ALLINI action.

The results presented here suggest several directions for future research. We were not able to crystallize the ALLINI GSK002 with IN, and the spectrum of resistance mutations elicited is mostly different between the two inhibitors. We speculate that the IN polymer formed in the presence of GSK002 is similar to that seen for GSK1264, with side chains remodeled to accommodate the interaction, but how these small structural differences influence inhibitor potency and escape mechanisms is just beginning to be studied. The role of the NTD in inhibition and escape is not yet understood but should also be experimentally accessible. Perhaps most importantly, our data suggest specific means of optimizing ALLINI binding in the observed interdomain pocket and specify regions of IN where additional functional groups may potentially be attached without disrupting binding. Thus, these results should be useful in optimizing the function of ALLINIs, a unique new class of HIV inhibitors that block viral replication by promoting inappropriate polymerization.

## Materials and Methods

### Ethics Statement

The human biological samples were sourced ethically from Gulf Coast Regional Blood Center (GCRBC). The protocol titled : IRB Specimen Procurement Protocol, Specimens to Provide to Internal or External Customers, was reviewed and approved by the GCRBC IRB (IRB approval # 06-001). The research conducted was in accord with the terms of the informed consents.

### Small Molecule Inhibitors

The compound GSK1264 was previously described [7] and is referred to as compound 159 in Patent WO 2012/102985 (Compound 159); GSK002 is described in Patent WO2013/012649 (Compound 87). Raltegravir-resistant strains HIV 4376 and HIV 8070 were obtained from the NIH AIDS repository.

### EM

A chronic HIV-1 producer cell line A1953 [45] was pretreated for 24 h using 100 and 1,000 nM GSK002, 100 and 1,000 nM GSK1264, or diluent control (DMSO). The cells were washed with 1 x PBS and incubated with fresh medium containing inhibitor or DMSO. After 72 h, the cells were harvested by centrifugation, and the cell pellets were fixed (2.0% paraformaldehyde, 2.5% glutaraldehyde in 0.2 M Na-CaCO, pH 7.3), embedded in Epon, and sectioned. Microscopy was performed with a JEM-1010 (JEOL, Tokyo, Japan) transmission electron microscope operated at 80 kV. Viral particles were categorized based on the viral morphology as mature, deformed (aberrant/eccentric/empty core), immature (no core), or ambiguous. For the 100 nM dose, findings were quantified by two blinded investigators.

### Protein Expression and Purification

Full-length and truncated HIV-1 IN(NL4-3) and coexpressions with LEDGF(IBD) (346–471) constructs were expressed and purified as described previously, with some modifications [46–48]. IN-only constructs encoding the F185H solubility mutation were expressed from a pETDuet-derived (Novagen) vector in which the IN construct was inserted in frame with a C-terminal Mxe intein (New England Biolabs) containing a chitin binding domain and hexahistidine tag. The QuikChange kit (Stratagene) was used to generate the point mutation. Proteins were purified using nickel-nitrilotriacetic acid (Qiagen) and chitin (New England Biolabs) resins. After fusion proteins were liberated by intein cleavage in 50 mM dithiothreitol (DTT) overnight at 4°C, IN preparations were further purified using a Superdex 75 HiLoad 16/60 column at room temperature, eluted isocratically in 20 mM HEPES-NaOH pH 7.0, 1 M NaCl, 7 mM CHAPS, 10 uM ZnOAc_2_, and 10 mM β-ME. Proteins were concentrated at 4°C in a YM-10 Centricon (Millipore), and aliquots were flash-frozen in liquid nitrogen for storage at −80°C. Genetically solubilized IN^C56S,F139D,F185H,280S^ (quadramutated, QM) with and without the additional W131D mutation (pentamutated, PM) were purified similarly into a final buffer of 20 mM HEPES-NaOH, pH 7.0, 450 mM NaCl, 7 mM CHAPS, 10 uM ZnOAc_2_, and 10 mM β-ME or 1–10 mM DTT. IN•LEDGF(IBD) coexpressions were produced from the same expression vector, except the IN-coding DNA was inserted into the first multiple cloning site (MCS) and LEDGF into the second MCS, in frame with the C-terminal Mxe intein-hexahistidine tag. These preparations were purified similarly into a final buffer of 20 mM HEPES-NaOH, pH 7.0, 1 M NaCl, 7 mM CHAPS, 10 uM ZnOAc_2_, and 10 mM β-ME or 1–10 mM DTT. IN^F185K^(CCD) used for crystallization was obtained by expression from the vector pET24 (Novagen, Madison, Wisconsin, United States) in BL21star (DE3) cells (Novagen) at 37°C. The sequence was inserted into the vector in frame with a N-terminal TEV-cleavable hexahistidine affinity tag. Protein was initially purified using Ni-Sepharose FF (GE Healthcare Life Sciences, Pittsburgh, Pennsylvania, US), followed by cleavage of the affinity tag using hexahistidine-tagged TEV at a 1:100 mass ratio in 25 mM HEPES-NaOH pH 7.5, 750 mM NaCl, and 25 mM Imidazole at 25°C with a 10K MWCO Jumbosep (Pall, Exton, Pennsylvania, US) centrifugal concentrator. To separate the liberated protein from uncleaved materials and protease, another Ni-Sepharose FF purification step was performed. The flow-through material was concentrated and injected onto a Superdex-75 column and eluted isocratically in 10 mM HEPES-NaOH pH 7, 500 mM NaCl, and 3 mM DTT. For biophysical analyses, samples were exchanged into 20 mM HEPES•NaOH pH 7.5, 1 M NaCl, 7 mM CHAPS, 10 mM DTT, and 10 μM Zn(OAc)_2_.

### Crystallization and Structure Determination

For crystallization, the inhibitor was prepared in 1,3-dimethyl-2-imidazolidinone (DMI, Fluka). Recombinant HIV IN^Y15A,F185H^ at 5–10 mg/mL concentrations in 20 mM HEPES pH 7.0, 0.5–1 M NaCl, 5 mM CHAPS, 10 μM ZnOAc_2_, 1–10 mM dithiothreitol (DTT), and 0.5–1.0 mM GSK1264 was mixed 1:1 with reservoir solutions containing 30% 2-methyl-2,4 pentanediol (MPD), 0.1 M sodium citrate pH 5.6–6.5 at 21°C in a standard hanging drop format. Crystals formed within 3–4 d and were iteratively improved by macroseeding. Diffraction data were obtained at the beam lines 5.0.2 and 12.3.1 of the Advanced Light Source (ALS, Berkeley, California, US).

All data reductions were performed using the program XDS [49]. Diffraction outwards of ~4 Å was observed in the best case, with resolution dropping off quickly as a function of X-ray dose. Analysis of the final dataset by the UCLA diffraction anisotropy server [50] indicated that diffraction was significantly anisotropic along the a*- and b*-axes. On the basis of an F/σ(F) cutoff of 3, reflections were subjected to an anisotropic truncation of 4.5, 4.5, and 4.3 Å along a*, b*, and c*, respectively, before use in refinement. Molecular replacement was performed using the program Phaser [51,52] as implemented in the Phenix software package [53]. Molecular replacement searches using a number of different combinations of monomer or dimer IN CCD^50-212^ (PDB 4OJR, [7]) and CTD^220-270^ (PDB 1EX4, [29]) all yielded strong molecular replacement solutions with very high LLG scores. In the initial experimental maps, strong and contiguous electron density was observed corresponding to the large helical extensions of IN^210:220^ seen in the 1EX4 crystal structure of IN(CCD-CTD) and was readily traced. After an initial iteration of simulated annealing, iterative rounds of rigid body refinement and minimization were performed in Phenix alongside manual building and refinement in COOT [54]. After an IN^50-270^ dimer was completely built, the structure was refined in CNS v1.3 [55,56] using rigid body refinement and the DEN method [26–28], using the high resolution IN^F185K^(CCD)•GSK1264 (PDB 4OJR, [7]) and residues 211–270 from the available 2.4 Å IN(CCD-CTD) (PDB 1EX4, [29]) structure as reference models. Ligand was omitted from the refinement until the latest stages. Strong ligand density corresponding to GSK1264 was observed in F_o_-F_c_ and simulated annealing F_o_-F_c_ omit maps, allowing for docking of the ligands at both binding sites at the IN(CCD) dimer interface. Composite omit maps were generated in Phenix, and figures created using PYMOL [57]. Crystallographic structures of IN(CCD)^F185K^ and related mutants were prepared and solved as previously described [7], using the beam line 21IDG at the Advanced Photon Source (Argonne, Illinois, US). Crystallographic statistics for the five structures are summarized in S2 Table. Atomic coordinates and structure factors were deposited in the Protein Data Bank under the accession codes 5HOT, 5HRN, 5HRP, 5HRR, and 5HRS.

### Virological Methods

Chronically infected HIV-1 producer A1953 cells were a gift from James Hoxie. The cells were cultured at 37°C in 5% CO_2_ in DMSO medium, supplemented with 10% fetal bovine serum and penicillin-streptomycin. To test the viability of HIV IN^Y15A^, DNA encoding the substitution was built into the HIV NL4-3 backbone using Gibson assembly. The sequence of the resulting plasmid was confirmed by Illumina sequencing of Nextera-XT libraries. Viral stocks (IN^Y15A^ and wild type) were generated by transfection into 293T cells and culture supernatants containing the HIV particles that were collected. Viral stocks were normalized by the amount of p24 capsid antigen per unit volume and then applied to TZMBL indicator cells. High luciferase activity was detected on day 2 after infection for wild type (2,334 RLU), whereas luciferase expression in IN^Y15A^ samples (21 RLUs) remained close to background (7 RLUs). A long-term culture experiment was carried out in which cells infected with the IN^Y15A^ virus were monitored for 2 mo by luciferase expression and p24 assay, but no viral replication could be detected.

### Virus Resistance Passage

MT4 cells infected with the HIV lab strain NL4-3 or Raltegravir-resistant viruses (NIH AIDS Research and Reference Reagent Program catalog #11842 and 11845) were cultured in the presence of suboptimal inhibitor concentrations at approximately one-half the IC_50_. Viral replication kinetics were monitored by the production of RT activity in the supernatant. When the kinetics of the inhibitor-treated culture matched that of the no-inhibitor control color for three consecutive passages, the inhibitor concentration was increased. At periodic intervals, approximately every ten passages, the virus was expanded, and IN sequence determined. Drug-induced mutations were created in the proviral plasmid pNL4-3 for confirmation of inhibitor sensitivity.

### HIV Replication Assays in PBMC Culture

Primary HIV-1 isolates were grown in human PBMCs. PBMCs were sedimented by layering blood obtained from donors over a Ficoll (Histopaque 1077; Sigma) cushion in 50 ml tubes. The cells were washed, pooled from various donors, and cryopreserved. PBMCs were thawed and stimulated with 2 μg/mL PHA in RPMI 1640 media supplemented with 10% heat-inactivated fetal bovine serum, 2 mM L-glutamine, 100 units/ml penicillin G, and 100 μg/ml streptomycin for 3 d prior to infection. Viral yields were determined for primary isolates 7 d post infection by a radioactive RT endpoint. To determine isolate sensitivity for various inhibitors, the appropriate titer of each isolate was used to infect PHA-stimulated PBMC pools in the presence of an inhibitor dilution series. Viral replication was measured by RT as the endpoint after 7 d in culture, and the IC_50_ calculated as the inhibitor concentration required to reduce viral replication by 50% of the control.

### Aggregation Assays

Assays were performed as previously described [7], with the following modification: turbidity assays were performed using VICTOR3V 1420 multilabel counter (PerkinElmer, Waltham, Massachusetts, US) by measuring the absorbance of the reaction solution at 405 nm. Final reaction conditions were 20 mM HEPES, pH 7.3, 375–505 mM NaCl, 3.75–5.05 mM CHAPS, 10 mM DTT, and 10 uM ZnAc_2_ with inhibitor concentrations ranging from 0.08 μM to 88 μM at 24–27°C. For the graphical representation of aggregation, the baseline (IN stock buffer + drug buffer) has been subtracted from the results.

### SEC-MALS

Absolute molecular weights were determined by multiangle light scattering coupled with refractive interferometric detection (Wyatt Technology, Santa Barbara, California, US) and a Superdex 200 10/300 GL column (GE Healthcare) at room temperature as previously described [48].

### Sedimentation Equilibrium Analysis

Sedimentation equilibrium AUC experiments were performed at 4°C with an XL-A analytical ultracentrifuge (Beckman-Coulter, Brea, California, US) and a TiAn60 rotor with two-channel charcoal-filled Epon centerpieces and quartz windows. Data were collected at 4°C with detection at 280 nm for 5, 7.5, and 10 μM samples. Linear analyses were performed by plotting the natural log of absorbance versus the square of radius, with the slope being proportional to *M*_*w*_. Single-species plots with calculated slopes for idealized oligomers were also calculated at a given speed for comparison.

### Oligonucleotide Assay for IN Activity

Assays for strand transfer were adapted from those described previously [58–60]. IN stocks were diluted into 20 mM HEPES•NaOH pH 7.5, 1 M NaCl, 7 mM CHAPS, 10 mM DTT, and 10 μM Zn(OAc)_2_ for a final concentration of 1 μM IN. Processed U5 LTR substrates with a 5′ Alexafluor 488 NHS Ester label were prepared by annealing the following oligonucleotides: 5′-Alexa488-ACCCTTTTAGTCAGTGTGGAAAATCTCTAGCA-3′ and 5′-TGACGATCTCTAAAAGGTGTGACTGATTTTCCCAG-3′ (IDT). Reactions were performed with 20 mM HEPES-NaOH pH 7.6, 0.5 μM Alexaflour 488-labeled LTR substrate, 15 nM pUC19 plasmid, 10 mM DTT, 100 mM NaCl, 5 mM MnCl_2_, 10 μM Zn(OAc)_2_, 5% DMSO, and 12% PEG-6000. Reactions were carried out for 60 min at 37°C and quenched using 0.5% SDS and 15 mM EDTA. After proteinase K treatment (1 mg/ml) for 30 min, reactions were separated on 1.5% Agarose gels in Tris-acetate buffer and visualized by EtBr staining or fluorescence using a Typhoon image station.

## Supporting Information

S1 Data Excel spreadsheet containing, in separate sheets, the underlying numerical data for Figure panels Fig 1C, Fig 4B–4H, Fig 5B–5D, Fig 7A, S1C–S1F Fig, S3B–S3D Fig and S5A Fig. (XLSX)

S1 Fig Properties of HIV-1 IN^Y15A,F185H^. (A) The D and E-forms of HIV IN(NTD) in isolation [22]. (B) The purification scheme and SDS-PAGE analysis of purified IN (32kD). Dissolved crystals of INY15A,F185H were washed and dissolved for SDS-PAGE analysis (right). (C) Sedimentation equilibrium analysis of IN^Y15A,F185H^. Data were recorded at 12,000 RPM, at a concentration of 10 μM IN, at 4°C. Linearized radial distributions are shown. The slopes are proportional to M_w_ at a given value of r^2^. Single-species plots with calculated slopes for idealized IN monomer, dimer, and tetramer are shown for the same rotor speed and temperature as grey lines. (D) SEC-MALS analysis of IN^Y15A,F185H^. The experiments were performed at room temperature using a Superdex 200 10/300 column. Samples were injected at 10 mg/mL. The elution concentrations by refractive index approached ~0.1 mg/mL. Both the M_w_ (weight-average molecular mass) from multiangle light scattering and retention times are consistent with a dimer of IN. (E) SEC-MALS analysis of IN^Y15A,F185H^•LEDGF(IBD). The M_w_ (weight-average molecular mass) from multiangle light scattering and retention times is consistent with a 2:2 LEDGF(IBD)-bound IN dimer. Data plotted in panels C, D, and E are provided in S1 Data. (F) ALLINI-induced aggregation of HIV-1 IN^Y15A,F185H^. (G) Crystals of HIV-1 IN^Y15A,F185H^ (top) and the corresponding X-ray diffraction from beam line ALS 5.0.3.
(TIF)

S2 Fig CHAPS binding at the IN CTD–catalytic core domain–1264 interface. (A) Superposition of the 1EX4 structure of IN CCD-catalytic core domain ([25], blue) superposed with the IN•GSK1264 crystal structure (grey) from this work. The structures superpose with a RMSD of 2.3 Å across all atoms of the CCD and catalytic core domain, differing mostly in the CTD regions, including the rotation of the SH3 domains relative to the catalytic core domain. CHAPS molecules observed in the 1EX4 structure are shown around the CTDs, rendered as blue sticks. (B) Overlay of the IN•GSK1264 structure with the 1EX4 CHAPS-bound model. Shown in red spheres is GSK1264, and in blue spheres, CHAPS. The position of 1264 and bound CHAPS coincide. (C) Electrostatic surfaces at the GSK1264–CTD–catalytic core domain interface. Cutaway views of the electrostatic surfaces at the CTD–catalytic core domain interface are shown. GSK1264 (ball and stick) is shown for reference. The interaction is bipartite, with packing between hydrophobic surfaces of α3 and β2 and complementary electrostatics between α5 and β5.
(TIF)

S3 Fig Effects of W131D on oligomerization and ALLINI-induced effects. (A) Approximately 80% of the buried interaction between CTD and catalytic core domain occurs with the α3 helix, and Trp131 is central to this packing. Highlighted here is Trp131 at this interface. (B) SEC-MALS analysis of IN-LEDGF coexpressions. Shown is a comparison of SEC-MALS data for a genetically solubilized IN background characterized previously (IN^C56S, F139D, F185H, C280S^; termed IN^QM^) [7,48,61] coexpressed with LEDGF(IBD). IN^QM^ •LEDGF(IBD) is a 4:2 complex in solution [48]. While the W131D mutation in this background retains LEDGF binding, mostly LEDGF-bound dimers are observed, consistent with the role of the residue at the CTD–catalytic core domain interface and the model presented in Fig 3. In complementary sedimentation equilibrium experiments (not shown), a K_d_ for dimer-tetramer of 90 μM is modelled for IN^QM-W131D^, versus 9 μM for control IN^QM^ at 4°C. (C) Sedimentation velocity analysis of IN^QM-W131D^. c(S) analysis of sedimentation velocity data is shown for full-length 30 μM IN^Q^ or IN^Q-W131D^ at 4°C and 20°C in the presence of DMSO (black) or 50 μM drug (grey). Species assigned as monomers (~1.8 S), dimers (~3.4 S), and tetramers (~5.5 S) are denoted. Distributions were derived from the fitting of the Lamm equation to the experimental data collected in the first 2 h of the experiment, as implemented in the program SEDFIT [62]. (D) Concentration and time-dependent aggregation of 9 μM IN^Q^ alone (upper left, [48]), 9 μM IN^P^ alone (lower left, this work), LEDGF-bound IN^Q^ (upper right, [48]), or LEDGF-bound IN^P^ (lower right, this work). The mutation W131D attenuates drug-induced aggregation of IN. Data plotted in panels B, C, and D are provided in S1 Data.
(TIF)

S4 Fig Structural basis for polymorphism-induced resistance to GSK002. Structural basis for polymorphism-induced resistance to GSK002. (A–C) Superposition of IN^F185K^(CCD) structures with GSK002 bound at the dimer interface. Shown in grey is IN^F185K^ bound with drug. Shown superposed are IN^A124T,F185K^ (green), IN^A124N,T125S,F185K^ (blue), and IN^A124N,T125A,F185K^ (yellow), all bound with GSK002. All structures superpose within 0.2 Å RMSD between α carbons. (D) Model of the GSK002-IN(CCD-CTD) drug binding interface. Highlighted are the predicted clashes between CTD, the difluorobenzyl moiety of GSK002, and residue 124.
(TIF)

S5 Fig Mutations at Y226 and W235 are inactive for strand transfer. (A) SEC-MALS analysis of IN^F185H^. Experiments were performed at room temperature using a Superdex 200 10/300 column. Data plotted here are provided in S1 Data. (B) Shown is a gel electrophoresis analysis of HIV IN mutants tested in strand transfer reactions involving preprocessed Alexaflour-488 labelled U5 substrate (35mers) mimicking the HIV LTR, as well as pUC19 plasmid (2.7 kB) serving as the integration target. Shown on the left is fluorescent imaging of products from the integration reaction and on the right the same gel visualized with ethidium bromide to show total DNA, which is predominately the plasmid target. While both half-site integrants (H.S.I) and full-site integrants (F.S.I) are seen for IN^F185H^, point mutants of Tyr226 (Y226A, Y226D, and Y226R) and W235 (W235A) are inactive for strand transfer over a 1-h time course.
(TIF)

S1 Table Solution properties of IN variants in this study. (DOCX)

S2 Table Summary of X-ray data processing and refinement statistics. (DOCX)

S3 Table Serial passage resistance data. (DOC)

## Abbreviations

ALLINI: allosteric inhibitor of integrase
ALS: Advanced Light Source
AUC: analytical ultracentrifugation
CHAPS: (3-((3-cholamidopropyl) dimethylammonio)-1-propanesulfonate)
CTD: C-terminal domain
DEN: deformable elastic network
EM: electron microscopy
HHCC: Histidine-Histidine-Cysteine-Cysteine motif
IBD: integrase binding domain
IN: integrase
INSTI: integrase strand transfer inhibitor
LEDGF/p75: lens epithelium-derived growth factor
NC: nucleocapsid
NCINI: noncatalytic site integrase inhibitors
NTD: N-terminal domain
SEC-MALS: size-exclusion chromatography and multiangle light scattering
SH3: Src homology domain 3
TPA: tetraphenylarsonium
