# Supplementary figures and images for: Structural Basis for Inhibitor-Induced Aggregation of HIV Integrase
Source: PLoS Biol. 2016 Dec 9;14(12):e1002584. doi: 10.1371/journal.pbio.1002584 (PMC5147827; doi:10.1371/journal.pbio.1002584)

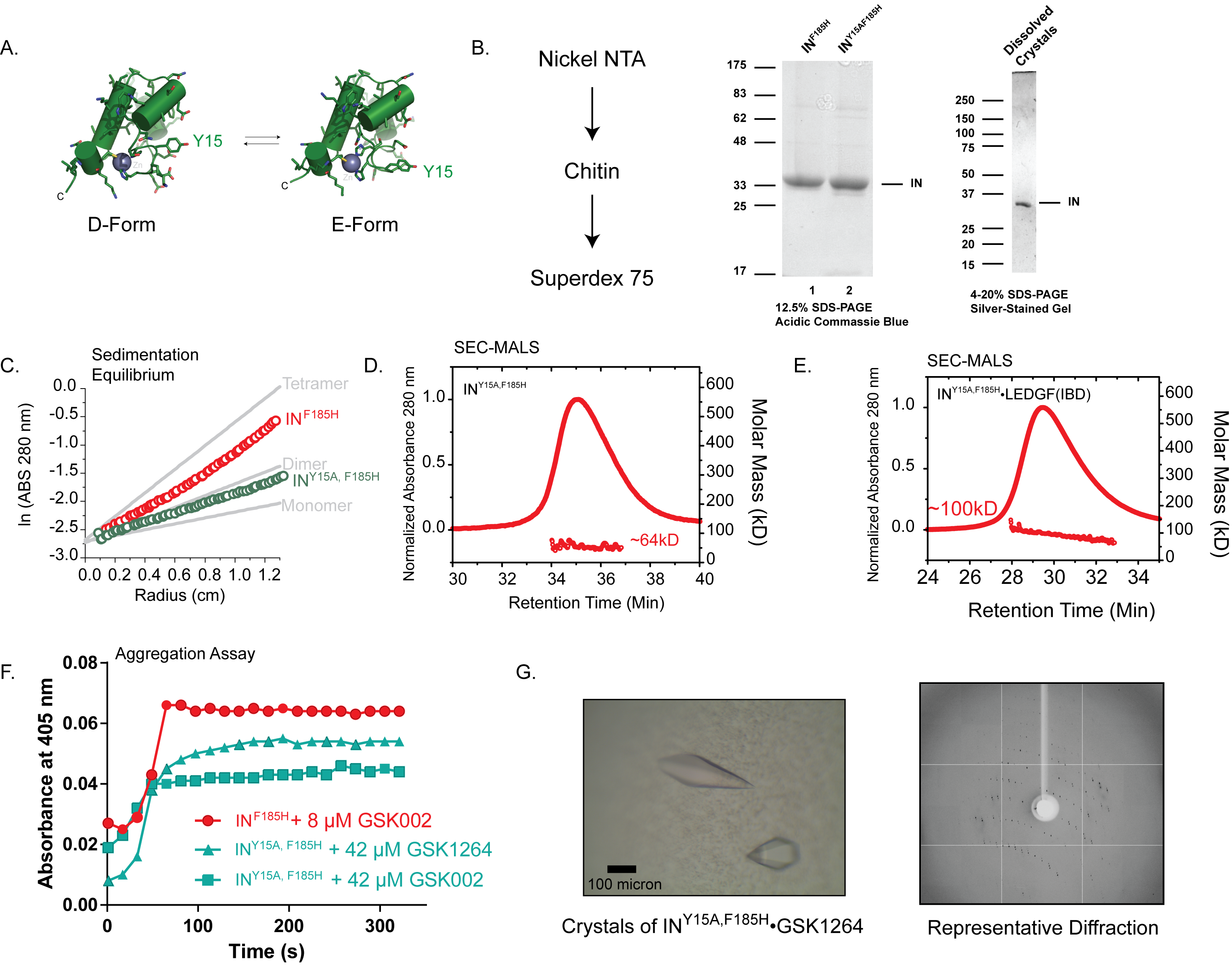

Supplement: S1 Fig — (A) The D and E-forms of HIV IN(NTD) in isolation [22]. (B) The purification scheme and SDS-PAGE analysis of purified IN (32kD). Dissolved crystals of INY15A,F185H were washed and dissolved for SDS-PAGE analysis (right). (C) Sedimentation equilibrium analysis of INY15A,F185H. Data were recorded at 12,000 RPM, at a concentration of 10 μM IN, at 4°C. Linearized radial distributions are shown. The slopes are proportional to Mw at a given value of r2. Single-species plots with calculated slopes for idealized IN monomer, dimer, and tetramer are shown for the same rotor speed and temperature as grey lines. (D) SEC-MALS analysis of INY15A,F185H. The experiments were performed at room temperature using a Superdex 200 10/300 column. Samples were injected at 10 mg/mL. The elution concentrations by refractive index approached ~0.1 mg/mL. Both the Mw (weight-average molecular mass) from multiangle light scattering and retention times are consistent with a dimer of IN. (E) SEC-MALS analysis of INY15A,F185H•LEDGF(IBD). The Mw (weight-average molecular mass) from multiangle light scattering and retention times is consistent with a 2:2 LEDGF(IBD)-bound IN dimer. Data plotted in panels C, D, and E are provided in S1 Data. (F) ALLINI-induced aggregation of HIV-1 INY15A,F185H. (G) Crystals of HIV-1 INY15A,F185H (top) and the corresponding X-ray diffraction from beam line ALS 5.0.3. (TIF) [file pbio.1002584.s002.tif]

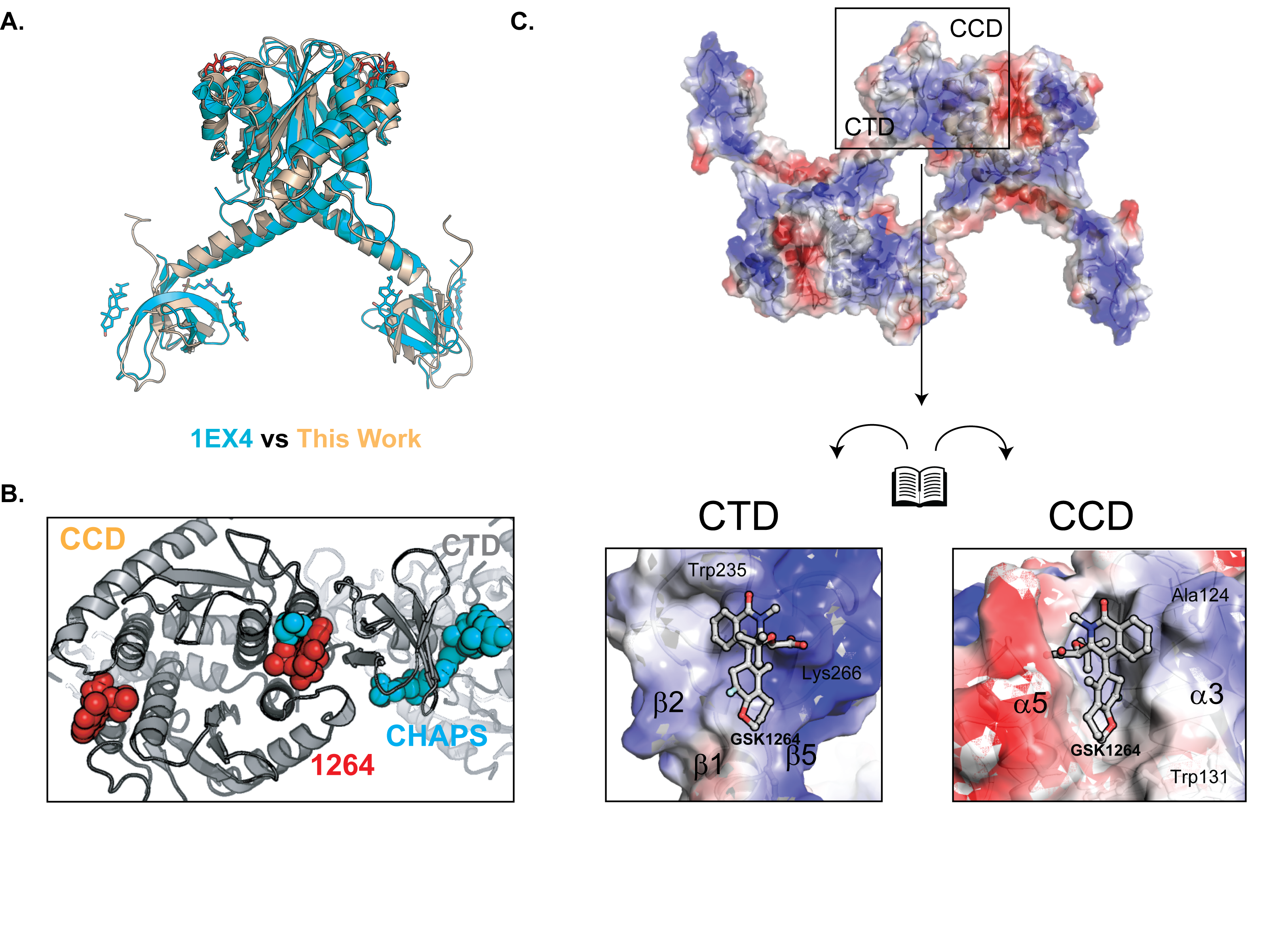

Supplement: S2 Fig — (A) Superposition of the 1EX4 structure of IN CCD-catalytic core domain ([25], blue) superposed with the IN•GSK1264 crystal structure (grey) from this work. The structures superpose with a RMSD of 2.3 Å across all atoms of the CCD and catalytic core domain, differing mostly in the CTD regions, including the rotation of the SH3 domains relative to the catalytic core domain. CHAPS molecules observed in the 1EX4 structure are shown around the CTDs, rendered as blue sticks. (B) Overlay of the IN•GSK1264 structure with the 1EX4 CHAPS-bound model. Shown in red spheres is GSK1264, and in blue spheres, CHAPS. The position of 1264 and bound CHAPS coincide. (C) Electrostatic surfaces at the GSK1264–CTD–catalytic core domain interface. Cutaway views of the electrostatic surfaces at the CTD–catalytic core domain interface are shown. GSK1264 (ball and stick) is shown for reference. The interaction is bipartite, with packing between hydrophobic surfaces of α3 and β2 and complementary electrostatics between α5 and β5. (TIF) [file pbio.1002584.s003.tif]

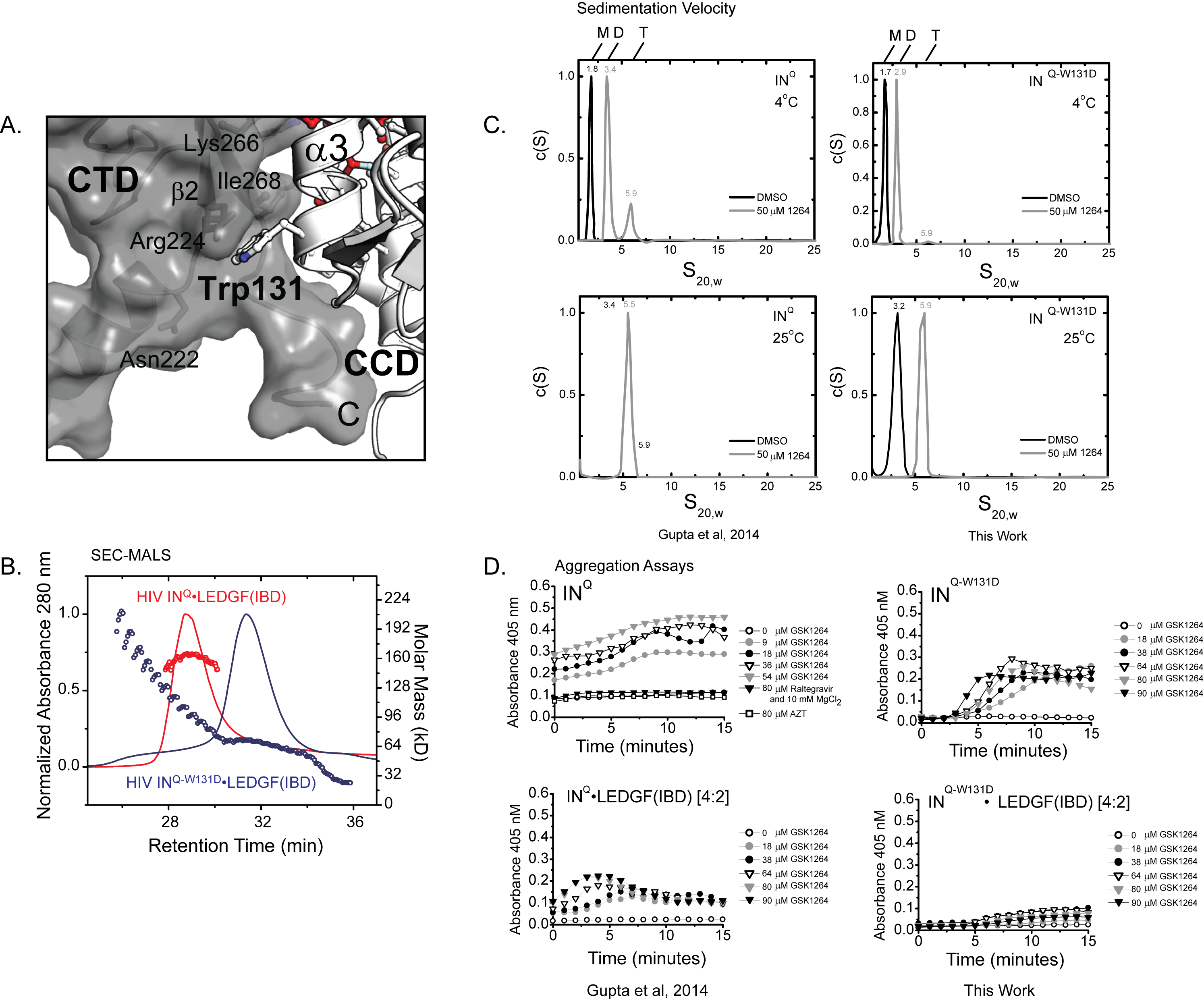

Supplement: S3 Fig — (A) Approximately 80% of the buried interaction between CTD and catalytic core domain occurs with the α3 helix, and Trp131 is central to this packing. Highlighted here is Trp131 at this interface. (B) SEC-MALS analysis of IN-LEDGF coexpressions. Shown is a comparison of SEC-MALS data for a genetically solubilized IN background characterized previously (INC56S, F139D, F185H, C280S; termed INQM) [7,48,61] coexpressed with LEDGF(IBD). INQM •LEDGF(IBD) is a 4:2 complex in solution [48]. While the W131D mutation in this background retains LEDGF binding, mostly LEDGF-bound dimers are observed, consistent with the role of the residue at the CTD–catalytic core domain interface and the model presented in Fig 3. In complementary sedimentation equilibrium experiments (not shown), a Kd for dimer-tetramer of 90 μM is modelled for INQM-W131D, versus 9 μM for control INQM at 4°C. (C) Sedimentation velocity analysis of INQM-W131D. c(S) analysis of sedimentation velocity data is shown for full-length 30 μM INQ or INQ-W131D at 4°C and 20°C in the presence of DMSO (black) or 50 μM drug (grey). Species assigned as monomers (~1.8 S), dimers (~3.4 S), and tetramers (~5.5 S) are denoted. Distributions were derived from the fitting of the Lamm equation to the experimental data collected in the first 2 h of the experiment, as implemented in the program SEDFIT [62]. (D) Concentration and time-dependent aggregation of 9 μM INQ alone (upper left, [48]), 9 μM INP alone (lower left, this work), LEDGF-bound INQ (upper right, [48]), or LEDGF-bound INP (lower right, this work). The mutation W131D attenuates drug-induced aggregation of IN. Data plotted in panels B, C, and D are provided in S1 Data. (TIF) [file pbio.1002584.s004.tif]

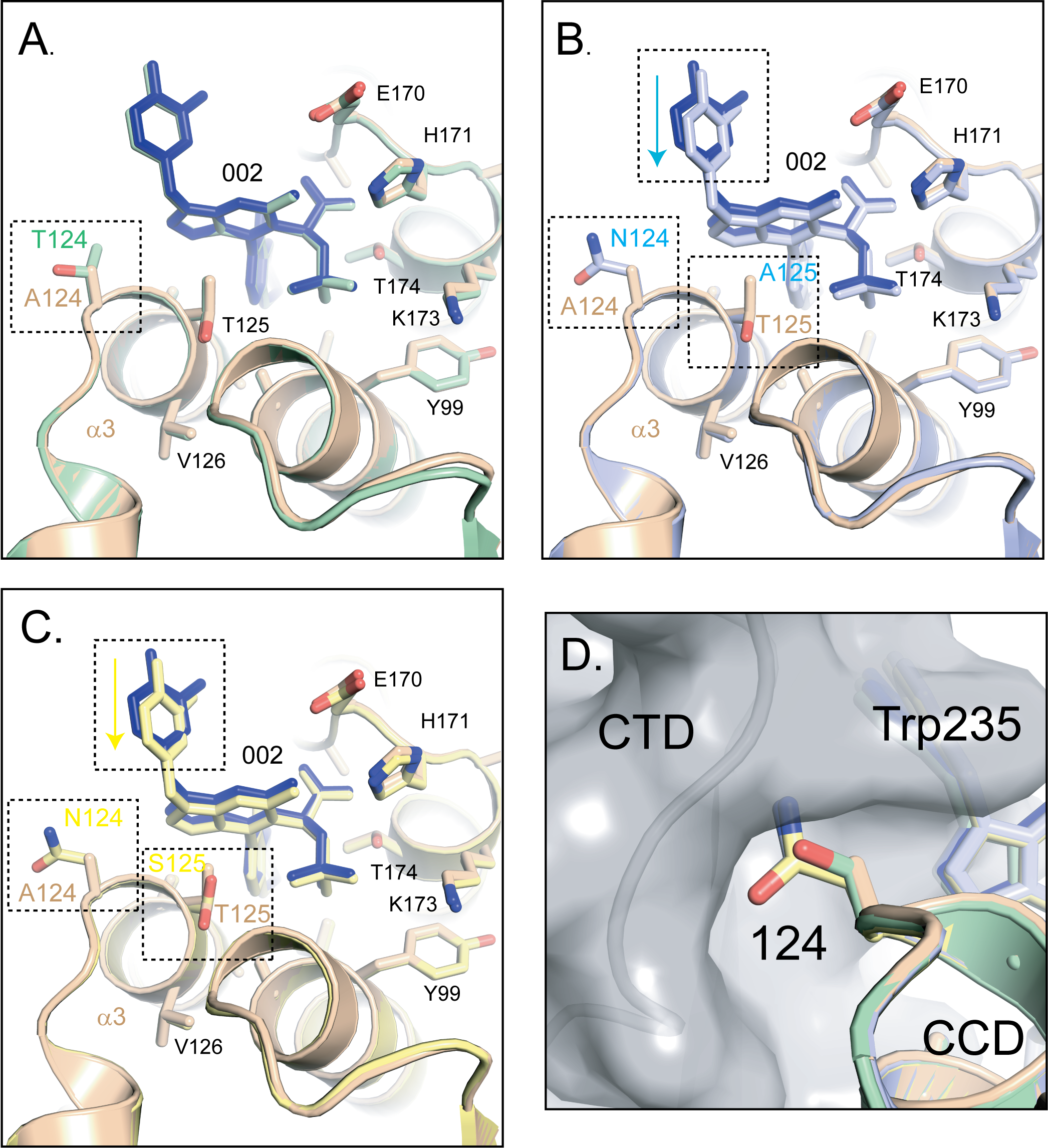

Supplement: S4 Fig — Structural basis for polymorphism-induced resistance to GSK002. (A–C) Superposition of INF185K(CCD) structures with GSK002 bound at the dimer interface. Shown in grey is INF185K bound with drug. Shown superposed are INA124T,F185K (green), INA124N,T125S,F185K (blue), and INA124N,T125A,F185K (yellow), all bound with GSK002. All structures superpose within 0.2 Å RMSD between α carbons. (D) Model of the GSK002-IN(CCD-CTD) drug binding interface. Highlighted are the predicted clashes between CTD, the difluorobenzyl moiety of GSK002, and residue 124. (TIF) [file pbio.1002584.s005.tif]

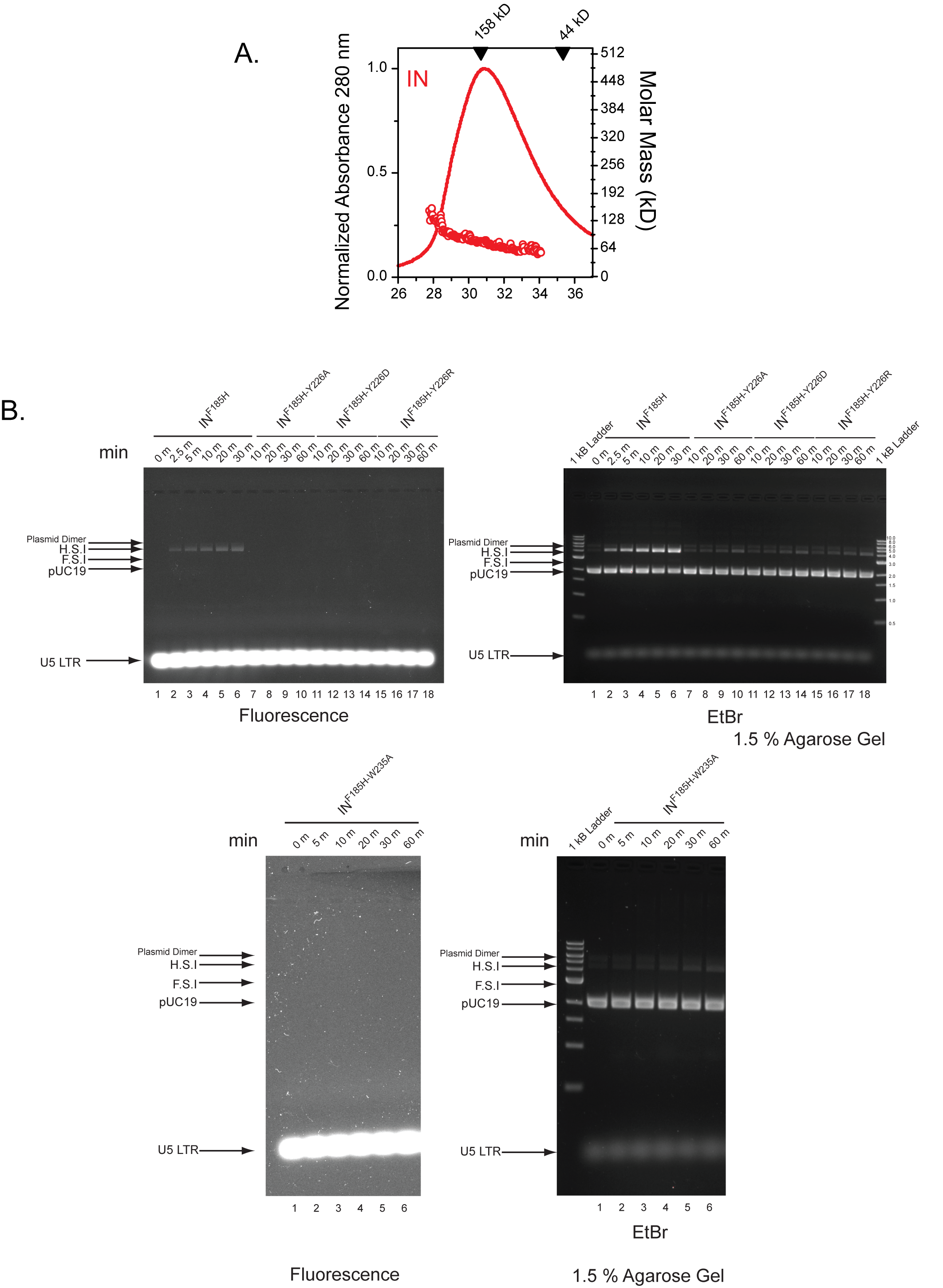

Supplement: S5 Fig — (A) SEC-MALS analysis of INF185H. Experiments were performed at room temperature using a Superdex 200 10/300 column. Data plotted here are provided in S1 Data. (B) Shown is a gel electrophoresis analysis of HIV IN mutants tested in strand transfer reactions involving preprocessed Alexaflour-488 labelled U5 substrate (35mers) mimicking the HIV LTR, as well as pUC19 plasmid (2.7 kB) serving as the integration target. Shown on the left is fluorescent imaging of products from the integration reaction and on the right the same gel visualized with ethidium bromide to show total DNA, which is predominately the plasmid target. While both half-site integrants (H.S.I) and full-site integrants (F.S.I) are seen for INF185H, point mutants of Tyr226 (Y226A, Y226D, and Y226R) and W235 (W235A) are inactive for strand transfer over a 1-h time course. (TIF) [file pbio.1002584.s006.tif]
