# Supplementary material for: Structural Basis for Inhibitor-Induced Aggregation of HIV Integrase
Source: PLoS Biol. 2016 Dec 9;14(12):e1002584. doi: 10.1371/journal.pbio.1002584 (PMC5147827; doi:10.1371/journal.pbio.1002584)
Supplement: S1 Table — (DOCX) [file pbio.1002584.s007.docx]

**S1 Table**

**Solution Properties of IN Variants in This Study.**

| Variants (IN^F185H^) | ALLINI Resistance Mutant? | Strand Transfer Activity^2^ (fmol/min) | Sedimentation Equilibrium (4^o^C)^3^ | SEC-MALS (Room Temperature)^4^ | *In vitro* Aggregation vs GSK1264 | *In vitro* Aggregation vs GSK002 |
| --- | --- | --- | --- | --- | --- | --- |
|  |  |  |  |  |  |  |
| IN^F185H^ | - | +++ | D<T | D<T | ++ | ++ |
|  |  |  |  |  |  |  |
| N-terminal domain mutations (NTD) |  |  |  |  |  |  |
| IN^D6R,F185H^ | Y | + | D>T | D-T | + | ++ |
| IN^E11K,F185H^ | N | - | *n.d.* | D>T | + | + |
| IN^Y15A,F185H^ | N | - | D | D | + | + |
|  |  |  |  |  |  |  |
| Catalytic Domain Mutations (CCD) |  |  |  |  |  |  |
| IN^A128T,F185H^ | Y | ++ | M-D | M-D+ | - | - |
| IN^W131C,F185H^ | Y | + | D<T | M-D | - | - |
| IN^QM^ | N | + | M-D-T^1^ | D-T^1^ | ++ | *n.d* |
| IN^W131D,QM^ | N | + | M-D | M-D | + | *n.d* |
| IN^T124A,W131C,F185H^ | Y | ++ | D-T | *n.d.* | + | + |
| IN^T124N,F185H^ | Y | ++ | D | *n.d.* | - |  |
|  |  |  |  |  |  |  |
| C-terminal Domain Mutations (CTD) |  |  |  |  |  |  |
| IN^F185H, N222K^ | Y | + | D>T | D>T | - | +/- |
| IN^Y99H,L172F, F185H, N222K,^ | Y | + | D | *n.d.* | +/- | + |
| IN^F185H, Y226A^ | N | - | M-D | D | + | + |
| IN^F185H, Y226D^ | N | - | D | D | - | +/- |
| IN^F185H, Y226R^ | N | - | D-T | D | - | +/- |
| IN^F185H, W235A^ | N | - | *Agg.* | *Agg.* | - | - |

1. ([Gupta et al., 2014](#_ENREF_11))
2. Strand transfer activity as determined by *in vitro* reactions analyzed by gel electrophoresis. ‘+++’ denotes > 90 fmol/min activity, ‘++’ denotes 10-90 fmol/min activity, ‘+’ denotes 1-10 fmol/min activity, and ‘-‘ denotes undetectable.
3. Oligomerization state was assessed by linear analysis of sedimentation equilibrium data recorded at 10-12,000 RPM with 10 μM IN at 4^o^C, relative to the calculated slopes for idealized IN monomer, dimer, and tetramer. M= monomer; D=dimer; T=tetramer. *n.d.* – Not determined. *Agg*. – aggregated.
4. Oligomerization states were categorized based on relative peak heights from SEC separation at room temperature together with measures M_w_ profiles across the half-peak. M= monomer; D=dimer; T=tetramer. *n.d.* – Not determined. *Agg.* – aggregated.
