# Supplementary material for: Structural Basis for Inhibitor-Induced Aggregation of HIV Integrase
Source: PLoS Biol. 2016 Dec 9;14(12):e1002584. doi: 10.1371/journal.pbio.1002584 (PMC5147827; doi:10.1371/journal.pbio.1002584)
Supplement: S2 Table — (DOCX) [file pbio.1002584.s008.docx]

**S2 Table**

**Summary of X-ray Data Processing and Refinement Statistics.**

| ***Data Collection*** | **IN(Y15A,F185H)**  **•GSK1264** | **IN^CCD^(F185K)**  **•GSK002** | **IN^CCD^(A124T,F185K)**  **•GSK002** | **IN^CCD^(A124N,T125S,F185K)**  **•GSK002** | **IN^CCD^(A124N,T125A,F185K)**  **•GSK002** |
| --- | --- | --- | --- | --- | --- |
| **PDB** | 5HOT | 5HRN | 5HRP | 5HRR | 5HRS |
| **Source** | ALS 5.0.2 | Rigaku FRE+ | APS 21IDF | APS 21IDF | APS 21IDF |
| **Resolution (Å)** | 20.0 – 4.4 | 18.1 – 1.75 | 18.1 – 1.81 | 17.7 – 1.88 | 18.1 – 1.75 |
| **Space Group** | P6_1_22 | P3_1_21 | P3_1_21 | P3_1_21 | P3_1_21 |
| **Unit-cell** | 107.06 Å x 107.06 Å x 243.49 Å  90^o^,90 ^o^,120^o^ | 72.67 Å x 72.67 Å x 65.44 Å  90 ^o^,90 ^o^,120 ^o^ | 72.82 Å x 72.82 Å x 65.87 Å  90^o^,90 ^o^,120 ^o^ | 72.60 Å x 72.60 Å x 65.72 Å  90^o^,90^o^,120^o^ | 72.52 Å x 72.52 Å x 65.69 Å  90 ^o^,90 ^o^,120^o^ |
| **Monomers in A.S.U.** | 2 | 1 | 1 | 1 | 1 |
| **No. of measured reflections** | 27,279 | 196,438 | 151,487 | 138,011 | 137,683 |
| **No. of unique reflections** | 5,416 | 21,401 | 20,804 | 18,964 | 18,851 |
| **Completeness (%)** | 93.9 (40.4) | 98.8 (98.8) | 99.9 (100.0) | 99.9 (100.0) | 99.8 (100.0) |
| **R_merge_ (%)^a^** | 6.5 (41.2) | 2.6 (44.3) | 6.1 (59.1) | 3.6 (65.4) | 5.3 (61.0) |
| **Mean I/σ(I)** | 17 (4.2) | 78.0 (3.1) | 25.5 (3.4) | 46.1 (2.8) | 30.8 (3.0) |
| **Multiplicity** | 5.0 (4.8) | 9.2 (4.9) | 7.3 (7.3) | 7.3 (7.3) | 7.3 (7.3) |
| **R_free_ (%)** | 36.0 | 22.5 | 22.5 | 23.5 | 21.5 |
| **R_factor_ (%)** | 31.2 | 18.7 | 18.7 | 19.46 | 19.94 |
| **R.M.S.D. bond angles (^o^)** | 0.007 | 0.010 | 0.010 | 0.010 | 0.010 |
| **R.M.S.D. bond lengths (Å)** | 1.30 | 0.88 | 0.88 | 0.93 | 0.92 |
| ***Ramachandran Most favored*** | 83.2 | 96.1 | 96.7 | 97.5 | 98.3 |
| ***Ramachandran Outlier*** | 0.3% | 0 | 0 | 0 | 0 |

R_merge_ = Σ |I_h_ - <I_h_>| / Σ I_h_, where <I_h_> is the average intensity over symmetry equivalent measurements

R-factor = Σ |F_obs_ – F_calc_| / Σ F_obs_, where summation is data used in refinement.

The summation for R_free_ was calculated with 10% of the data for the first structure, 3.2% for the CCD structures.

Numbers in parentheses represent values in the highest-resolution shell.
