# Supplementary material for: Structural Basis for Inhibitor-Induced Aggregation of HIV Integrase
Source: PLoS Biol. 2016 Dec 9;14(12):e1002584. doi: 10.1371/journal.pbio.1002584 (PMC5147827; doi:10.1371/journal.pbio.1002584)
Supplement: S3 Table — (DOC) [file pbio.1002584.s009.doc]

**S3 Table**

**Serial Passage Resistance Data**

|  |  | **IC_50_** | **NL4-3** | **IC_50_** | **4376** | **IC_50_** | **8070** |
| --- | --- | --- | --- | --- | --- | --- | --- |
| **Compound** | **Passage #** | **[μM]** | **Sequence** | **[μM]** | **Sequence** | **[μM]** | **Sequence** |
| **GSK2880002A** | 8 | 0.005 | W131C | 0.001 | E6R | 0.022 | A128X |
|  | 18 | 0.160 | W131C,A205T | *n.d.* |  | *n.d.* |  |
|  | 31 | 0.640 | T124N, W131C, A205T | *n.d.* |  | *n.d.* |  |
|  | 38 | 0.640 | T124N, W131C, A205T | *n.d.* | E170K, K173N | *n.d.* | T124N, H171Q |
|  |  |  |  |  |  |  |  |
| **GSK2791264A** | 8 | 0.06 | Y99H, N222K | 0.004 | NC | 0.106 |  |
|  | 18 | 0.20 | Y99H, N222K | *n.d.* |  | *n.d.* |  |
|  | 31 | 0.80 | Y99H, L172F, N222K | *n.d.* |  | *n.d.* |  |
|  | 38 | 0.80 | Y99H, L172F, N222K | *n.d.* | A129T, A205T | *n.d.* | T174I |

Three strains of HIV-1 were passaged in the presence of increasing concentrations of each inhibitor and the resulting resistant viral strains isolated and genotyped. Three different HIV-1 strains were compared for each inhibitor: the lab-adapted HIV strain NL4-3 and Raltegravir-resistant strains 4376 and 8070. Strains were passaged 38 times in the presence of compound, resulting in an increase in the inhibitory IC_50_ of >100-fold. IN coding regions were sequenced and departures from wild-type sequence tabulated

*n.d.* – Not Determined.
